# Flexible photonic crystal membranes with nanoparticle high refractive index layers

**DOI:** 10.3762/bjnano.8.22

**Published:** 2017-01-20

**Authors:** Torben Karrock, Moritz Paulsen, Martina Gerken

**Affiliations:** 1 Institute of Electrical Engineering and Information Technology, Christian-Albrechts-Universität zu Kiel, Kaiserstr. 2, Kiel, 24143, Germany

**Keywords:** fabrication, flexible membrane, nanostructure, photonic crystal, resonance shift

## Abstract

Flexible photonic crystal slabs with an area of 2 cm^2^ are fabricated by nanoimprint replication of a 400 nm period linear grating nanostructure into a ≈60 µm thick polydimethylsiloxane membrane and subsequent spin coating of a high refractive index titanium dioxide nanoparticle layer. Samples are prepared with different nanoparticle concentrations. Guided-mode resonances with a quality factor of *Q* ≈ 40 are observed. The highly flexible nature of the membranes allows for stretching of up to 20% elongation. Resonance peak positions for unstretched samples vary from 555 to 630 nm depending on the particle concentration. Stretching results in a resonance shift for these peaks of up to ≈80 nm, i.e., 3.9 nm per % strain. The color impression of the samples observed with crossed-polarization filters changes from the green to the red regime. The high tunability renders these membranes promising for both tunable optical devices as well as visualization devices.

## Introduction

Photonic crystal slabs (also called resonance waveguide gratings) consist of a guiding layer with high refractive index on a nanostructured substrate. A subwavelength grating gives the incident light a lateral momentum and thus allows the coupling from incident waves to quasi-guided modes and vice versa [1–2]. Photonic crystal slabs feature guided mode resonances (GMRs) which are observed as dips or peaks with a Fano line shape in the transmission and the reflection spectrum [3]. The resonance wavelength depends on the gratings properties, the waveguide properties, and the angle of incidence. Many different fabrication approaches have been presented and recent publications show high potential for future products, including multiparametric label-free biosensing [4], photonic crystal enhanced microscopy [5], single molecule trapping [6], and surface emitting lasers [7]. Recently, flexible photonic crystal structures with elastomers as substrates have been investigated as strain sensors [8], for enhanced light out-coupling in flexible organic light emitting diodes [9–10], for photonic paper [11], and for pressure sensing [12]. N. L. Privorotskaya et al. [13] demonstrated a resonance shift of 4.53 nm per % strain and S. J. Foland and J.-B. Lee [14] measured 4.8 nm per % strain. The samples were stretched up to 3.75% and 5% strain, respectively. We previously identified cracking of continuous high-index waveguide layers as a limitation for the performance of flexible photonic crystals and demonstrated a nanoparticulate high-index layer [15]. Our previous study [12] showed first promising results in the use of flexible photonic crystals. Therefore, we conducted an in-depth study of flexible photonic crystal membranes with varying nanoparticle concentrations and different grating properties. Here, we present detailed results of the fabrication and optical properties of these flexible photonic crystals membranes with nanoparticulate high-index layers that allow for strain values of up to 20%.

**Figure 1:**
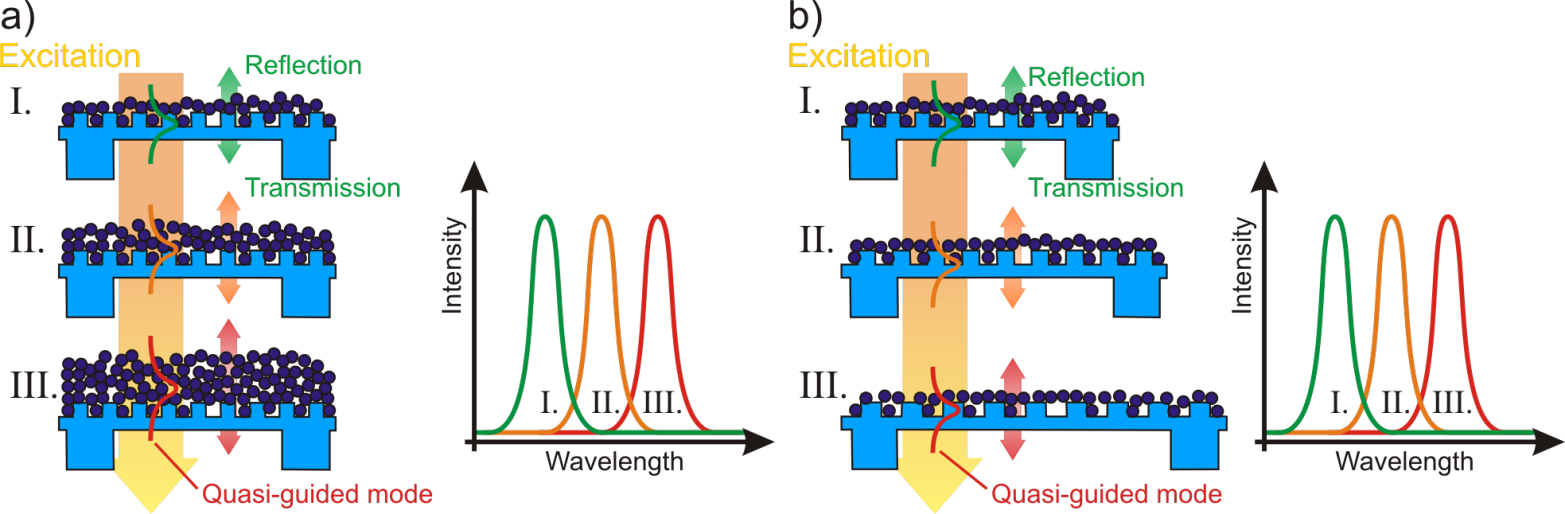
A 60 µm thick polydimethylsiloxane (PDMS) membrane with a 400 nm period and 140 nm deep linear grating nanostructure is coated with a high-refractive index layer of titanium dioxide (TiO_2_) nanoparticles. Resonances are observed in the normal-incidence transmission spectrum between crossed-polarization filters. The resonance shift is investigated as a function of a) the nanoparticle layer thickness and b) strain. (Structure dimensions not to scale).

We fabricate these highly flexible photonic crystal slabs by utilizing nanoreplication of a linear grating nanostructure with a period of 400 nm into a polydimethylsiloxane (PDMS) membrane and subsequent spin coating of titanium dioxide (TiO_2_) nanoparticles. Investigations with 300 nm and 500 nm gratings lead to similar results only shifted to respectively lower and higher wavelength ranges. Therefore, we decided to use only the 400 nm grating for this detailed study on the influence of high-index layer thickness and elongation behavior. The thickness of the high-index layer is varied by employing different concentrations of nanoparticles in distilled water, ranging from 2 wt % to 12 wt %. The experimental results are presented for the spectral properties of six different nanoparticle layer thicknesses and for strain values from 0% to 20%. As depicted schematically in Figure 1 we expect a spectral shift of the resonance both with nanoparticle layer thickness and with strain. Due to the large possible deformations and color changes such photonic crystals membranes are highly promising for tunable devices as well as for visualization devices.

## Results and Discussion

**Figure 2:**
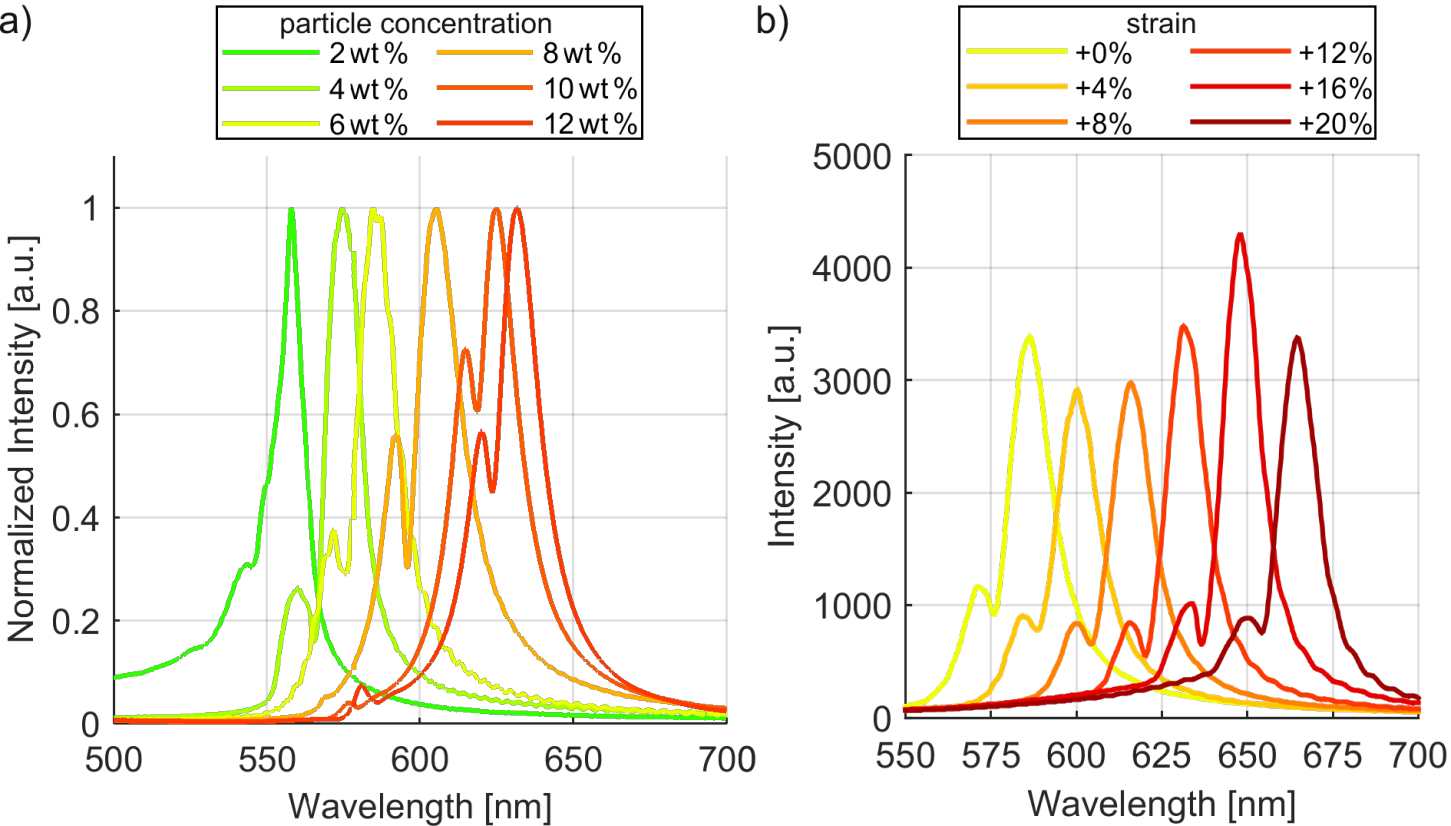
Resonance spectra with crossed polarization filters at a) 0% strain for samples with 2 wt % to 12 wt % TiO_2_ and at b) 6 wt % TiO_2_ sample for strain values of +0 to +20% perpendicular to the grating direction. A resonance shift from 586 to 664 nm is obtained corresponding to 3.9 nm/% strain.

We observe resonances in the transmission spectrum for all nanoparticle concentrations from 2 wt % to 12 wt % demonstrating that the particles form a waveguide layer. Below 2 wt % no resonances are detected suggesting that the waveguiding is not sufficient. At 12 wt % the resonances started to leave the visible spectrum. A test sample with 14 wt % concentration exhibits a poor quality of resonances that could not be used for meaningful statements. Figure 2a shows the spectra for the different nanoparticle concentrations. The resonances exhibit a quality factor *Q* of ≈40. The resonance wavelength shifts with the nanoparticle concentration.

This is expected as a higher nanoparticle concentration results in a thicker nanoparticle layer and thus a higher effective refractive index *n*_eff_ of the mode. The wavevector of the mode *k*_mode_ = *n*_eff_*k*_0_ is related to the grating vector *k*_Gx_ = 2π/Λ by Equation 1 [15].

[1]
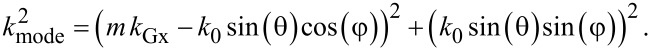


*k*_0_ = 2π/λ is the vacuum-wavevector, λ is the resonance wavelength in vacuum, Λ is the grating period, *m* is the resonance order, θ is the incidence angles to the surface normal, and φ is the angle between the grating lines and the projection of the incidence light onto the surface [15]. In our setup we use φ = 0° and θ = 0°. Therefore, Equation 1 reduces to:

[2]
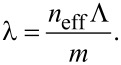


Using Equation 2 we calculate the effective refractive index *n*_eff_ of the mode for the different nanoparticle concentrations from the period Λ = 400 nm, resonance wavelength λ, and the order *m* = 1. The values are summarized in Table 1. The quality factor (*Q*) given in Table 1 is calculated with Equation 3 and the data from the spectra in Figure 2a.

[3]
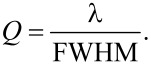


FWHM (full-width half-max) is the resonance peak’s width at 50% of its maximal intensity. *Q* for the 10 wt % sample is not evaluated with this equation as a second peak that is higher than 50% of the main peak’s intensity overlaps the main peak.

**Table 1:** *n*_eff_ and *Q*-factor values with respect to the concentration of TiO_2_ nanoparticles in the spincoat solution. *n*_eff_ is calculated with Equation 2 and *Q* is calculated with Equation 3.

Particle concentration	*n*_eff_	*Q*

2 wt %	1.435	46
4 wt %	1.483	44
6 wt %	1.514	36
8 wt %	1.564	35
10 wt %	1.611	double peak
12 wt %	1.634	42

**Figure 3:**
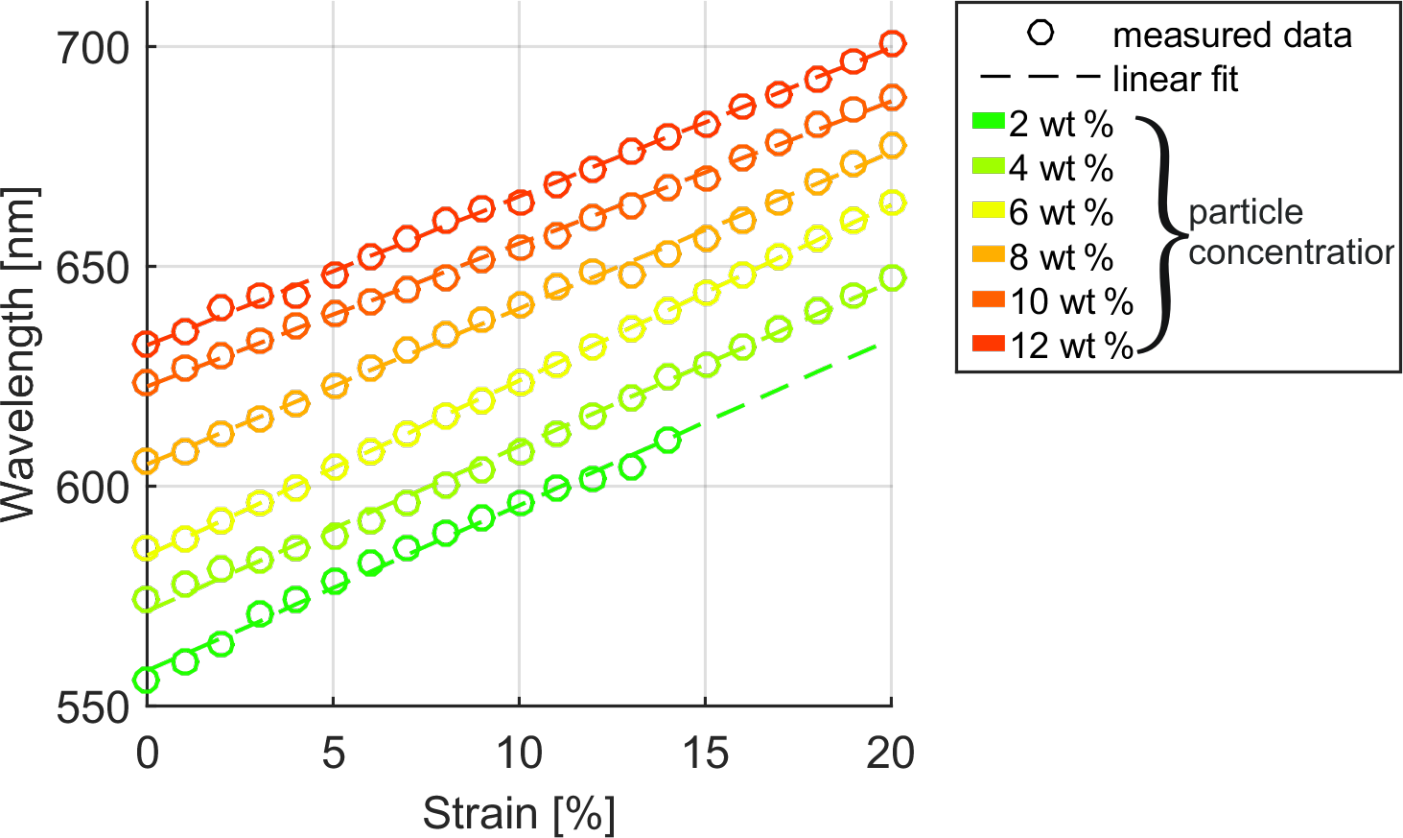
The shift of the resonance wavelength for 6 different samples with particle concentrations in the dilution from 2 to 12 wt % are shown for 0 to 20% strain. All resonance wavelengths rise for increasing particle concentration and increasing strain.

Next, the samples are stretched from 0% to 20% of additional length in 1% steps. Figure 2b shows example strain spectra of the 6 wt % nanoparticle sample. Stretching the samples leads to an increase of the period Λ as well as a decrease of *n*_eff_ as the nanoparticles are distributed across a larger surface. As seen from Equation 2 these two changes have opposite effects on the resonance wavelength λ. Additional, the effective refractive index for modes of higher wavelength reduces due to the smaller confinement factor in the waveguide layer. Figure 3 plots the experimentally observed resonance shifts under strain. The experimental data shows on the example of the 6 wt % resonance that a 20% strain results in negligible decrease of the quality factor and a wavelength shift from 586 to 664 nm. This corresponds to 3.9 nm/% strain. This shift with strain is on the same order as values found in literature, but operation for a significantly larger total strain of 20% is demonstrated compared to 5% in previous work [13–14].

As shown in Figure 3 the resonance shift is nearly linear with strain for all samples. To illustrate this, a linear fit is also given in the figure for every sample. For the 2 wt % sample measurement points from 15 to 20% could not be acquired because the sample ruptured at 15%. In this sample, air bubbles were trapped in the PDMS substrate because of insufficient degassing in the fabrication. Due to the air bubbles, the PDMS substrate was significantly thinner and could not withstand the strain above 15%.

**Figure 4:**
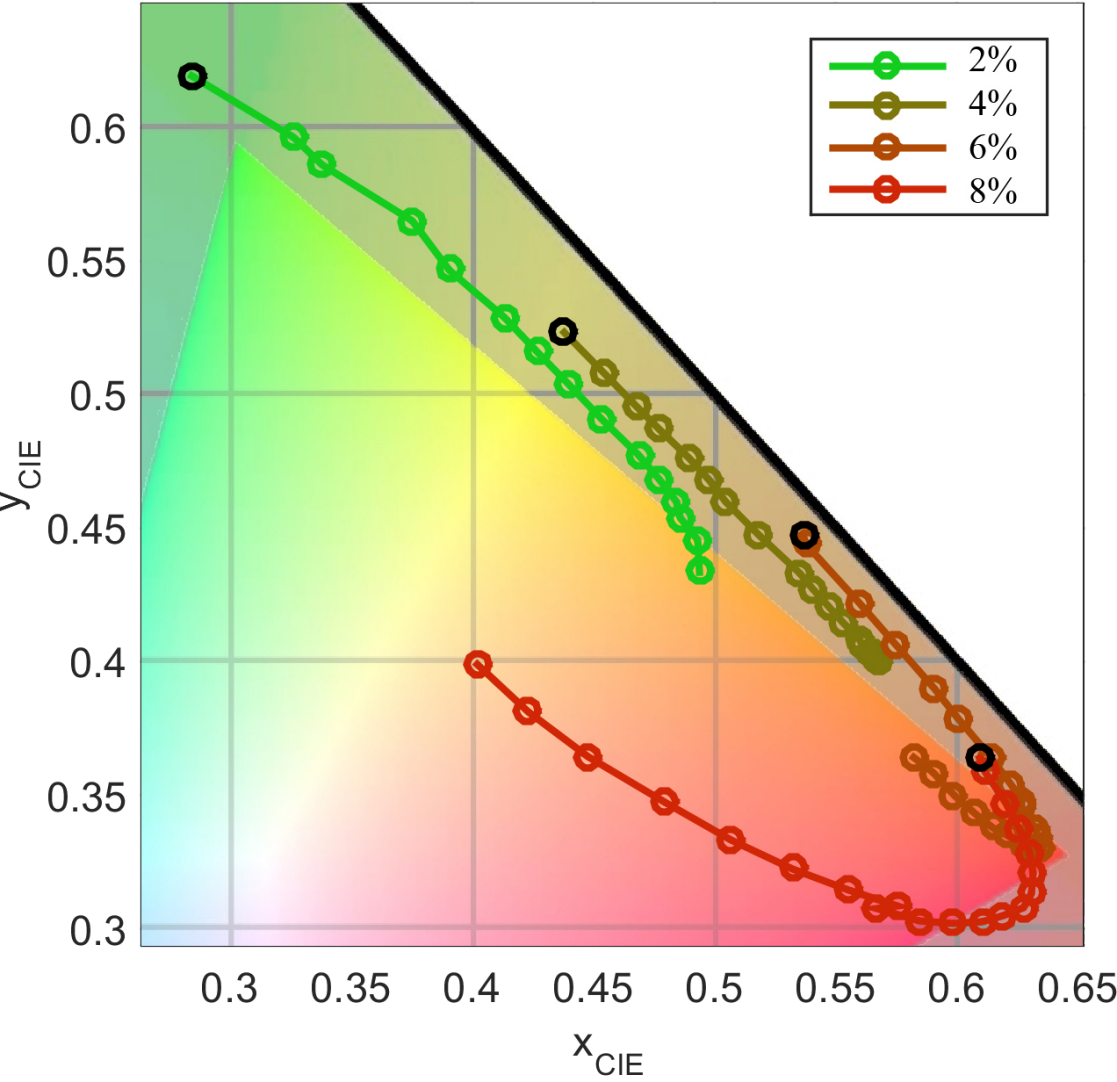
CIE values of the spectra of four different samples (2 to 8% TiO_2_) for strain values of 0 to 20% perpendicular to their grating direction. The measurements form lines parallel to the spectral locus from green to red (the black circle is the starting point with 0% strain for each sample).

The large observed resonance shifts of ≈80 nm correspond to significant color shifts in the visual spectrum. In order to investigate the visualization aspect more closely, the measured spectra are converted to the standard CIE color system. Figure 4 shows the converted curves for the samples from 2 to 8 wt % of nanoparticles. The spectra of higher concentrations are not shown as their resonances lie outside the standard CIE observer’s sensitivity curves and thus, only the background noise is converted to the CIE system. The broad background noise gives a white color impression. This effect starts to be visible in the curve of the 6 and 8 wt % curves and leads to a bending of the curve towards the white point in the CIE coordinate system. The other curves are nearly parallel to the locus curve of the color system and in its vicinity. This corresponds to relatively sharp resonances approaching the ones of monochromatic lasers which form the locus curve.

**Figure 5:**
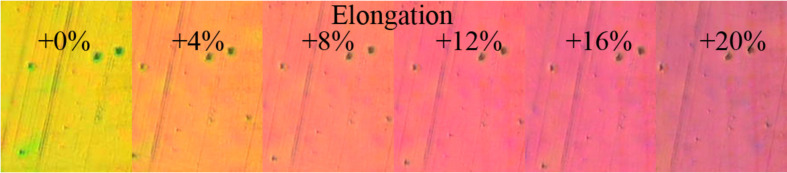
Photographs of the 6 wt % TiO_2_ sample at strain values of 0 to 20% taken with crossed polarization filters.

The curves in Figure 4 predict color changes from green to red for the photonic crystal membranes. As an example for this color change, Figure 5 shows photographs for the 6 wt % nanoparticle sample taken with crossed polarization filters for strain values from +0 to +20%. A color shift from yellow to red is achieved as predicted from the CIE color curve.

## Conclusion

We investigated the tunability of flexible photonic crystal membranes fabricated by nanoreplication of a linear grating master into a ≈60 µm thick membrane and subsequent spin coating of a TiO_2_ nanoparticle waveguide layer. Using a 400 nm period linear grating nanostructure, a single resonance peak in the visible spectrum is obtained. The influence of the particle concentration on the resonance peak position was examined and the optical characteristics for different samples during elongation were investigated. The resonance wavelength is adjustable from 555 to 630 nm during the fabrication process by varying the concentration of nanoparticles in the solution used for spin coating. The photonic crystal membranes were stretched perpendicular to the grating direction up to additional 20% of their original length. This induces a shift of ≈80 nm in the resonance wavelength. This resonance shift corresponds to a significant color change in the CIE color system when observed with crossed polarization filters. The flexibility of the photonic crystal membranes and the large possible strain values could lead to new applications in visualization devices such as remote-readout strain gauges for large strain values. For example, mounting one of the 2 cm^2^ photonic crystal membranes to a flexible device under test could allow for the evaluation of local strain values with optical resolution by simple evaluation of the color impression on photographs.

## Experimental

The fabrication of the flexible photonic crystal is performed in two steps. First a thin, nanostructured PDMS membrane with a refractive index of *n* = 1.42 is fabricated by a nanoimprint step. In the second step, high refractive index TiO_2_ nanoparticles are deposited onto the membrane by spin coating to create the waveguiding layer of the photonic crystal slab.

### Nanoimprint of PDMS membranes

Nanoimprint is an easy, low-cost fabrication technique for high throughput and high resolution replication of large area nanostructures. PDMS (Sylgard 184 PDMS by Dow Corning, Midland, MI, USA) is used as the membrane material. It consists of a base polymer (part A) and a curing agent (part B). PDMS received broad attention for the fabrication of microsystems such as microfluidic and lab-on-a-chip systems [16]. It is non-toxic, biocompatible and may be used for mass production in medical applications [17–18]. The mixture is cured by an organometallic cross-linking reaction that is promoted by heat resulting in an amorphous network of polymer chains. The random PDMS coils are 0.7 nm in diameter and have a dimension of about 10 nm [16]. Thus, a contour accuracy of around 10 nm is possible. Its transparent nature and refractive index (given by the vendor as 1.41 at 589 nm) renders it suitable for our optical applications.

While PDMS has many valuable features, in our case its hydrophobicity is an obstacle for a later water-based fabrication step. The water contact angle of untreated PDMS is 105–115° [19]. To alter the hydrophobic surface of PDMS an O_2_ plasma treatment is commonly used to temporarily create a hydrophilic surface [17,19]. To prevent damage to the nanostructure we instead add poly(dimethylsiloxane)-b-poly(ethylene oxide) (PDMS-b-PEO by Polysciences, Hirschberg an der Bergstrasse, Germany) to the uncured mixture of the base part and curing agent. It comprises a hydrophilic anchor, which is compatible with the base elastomer and hydrophilic pendant chains. With the adsorption and reorientation of the PEO to the surface–water interface the interfacial free energy is reduced. The modified PDMS surface exhibits a time-dependent water contact angle. It drops rapidly in the first 30 s and is stable after ≈200 s. The contact angle is reduced to 20° at a concentration of about 2% of PEO in the PDMS mixture. A side effect of mixing PDMS-b-PEO with PDMS is a reduced transparency, but due to our thin membrane structure this effect is not important [19].

**Figure 6:**
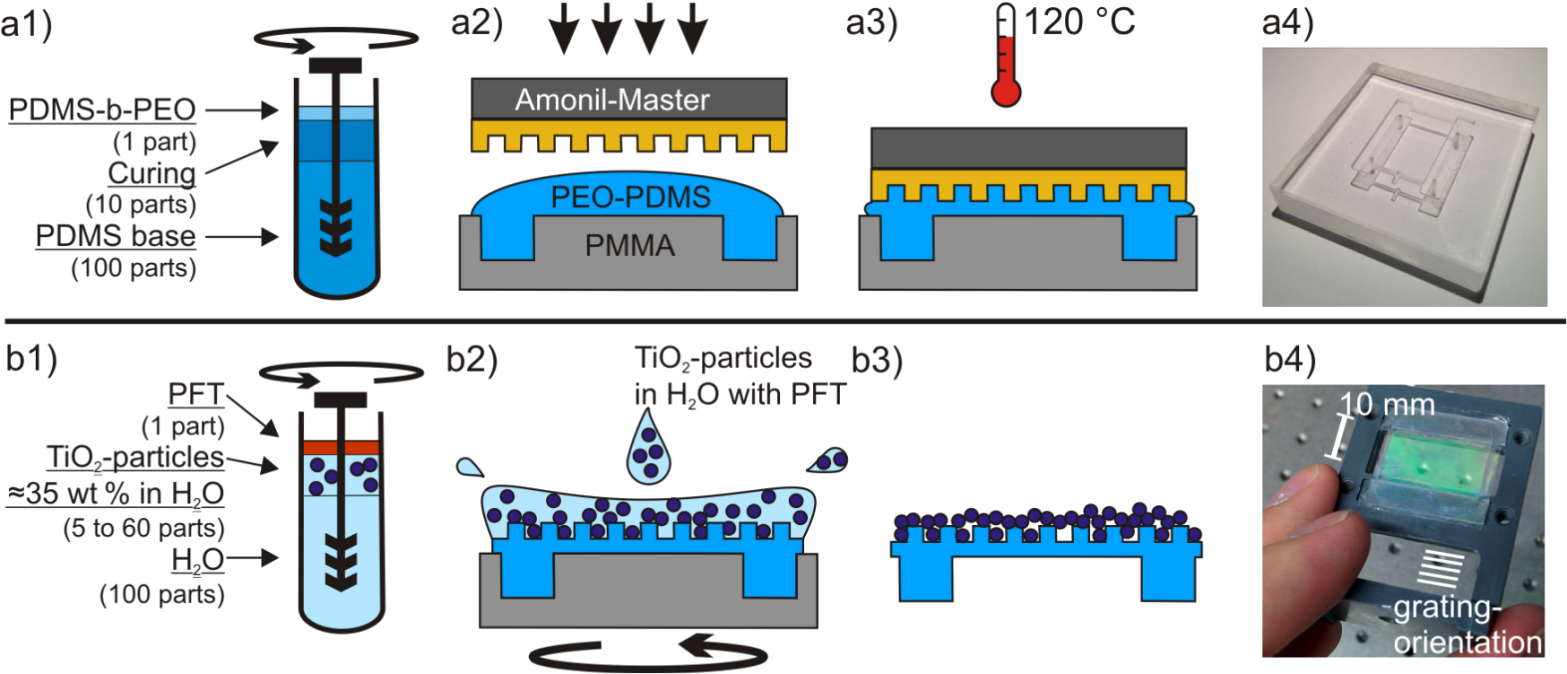
Fabrication of the flexible photonic crystal membrane by molding and imprinting a PDMS membrane with a nanostructure (a1–a3) and applying a high index layer by spin coating of high index nanoparticles (b1–b3). a4) shows the mold for the PDMS substrate and b4) shows a completed photonic crystal membrane on which the grating reflections are visible.

The PDMS base part, the curing agent, and the PDMS-b-PEO are mixed in a ratio of 100:10:1. This leads to a rather soft and flexible cross-linked polymer with reduced surface hydrophobicity. The mixture is thoroughly stirred for 5 min at 500 rpm (IKA ULTRA-TURRAX Tube Drive). The mixture is degassed in vacuum for 30 min. The mixed PDMS is poured into a poly(methyl methacrylate) (PMMA) mold which defines the form and size for the photonic crystal membrane to be created. It is allowed to flow in the indentations by gravity. On top a nanostructured photo resist stamp is pressed. This secondary stamp was replicated in Amonil (AMO GmbH, Aachen, Germany) from an electron-beam written glass master with a linear grating period of 400 nm and a grating depth of 140 nm (AMO GmbH, Aachen, Germany) in a lithographic nanoimprint process. Details on the process are given in [4]. As only the bearing structures that support the flexible photonic crystal membrane are milled out of the PMMA mold, the main part of the mold has a smooth surface. When placing the Amonil stamp on the overly filled mold, not all of the PDMS flows out and a thin film that later forms the ≈60 µm thick membrane remains. The filled PMMA mold with the nanostructure stamp on top is heated at 120 °C for 45 min (Figure 6a1–6a3). Figure 6a4 shows a picture of the PMMA mold that is used to create the supported membrane.

### Spin coating of the high index layer

**Figure 7:**
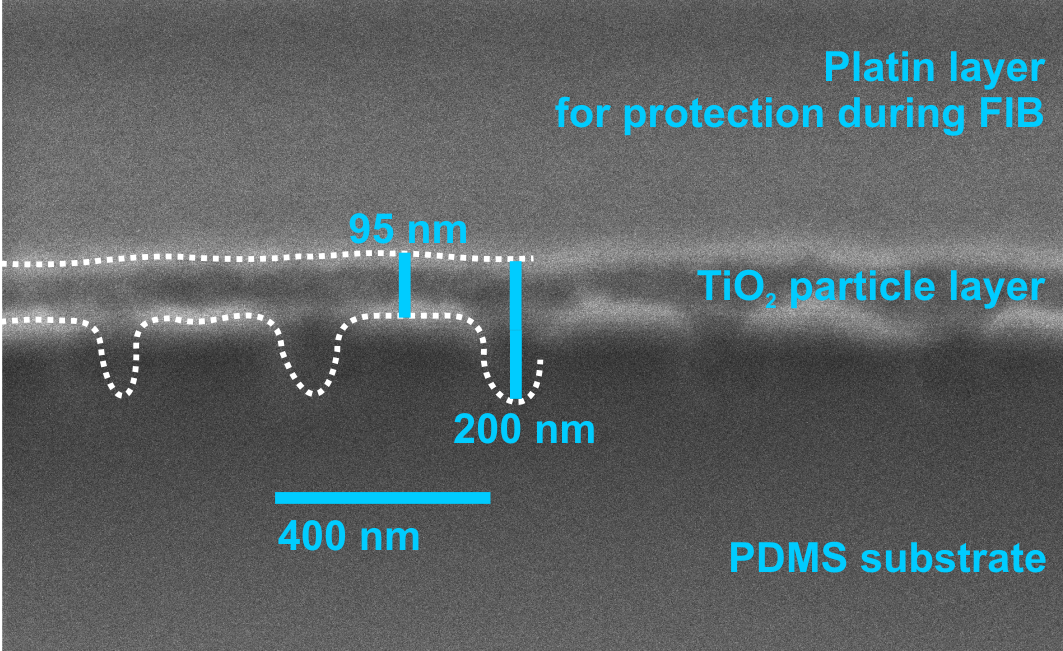
Cross-section of a 400 nm grating on a PDMS membrane with a particle layer created by a 6 wt % solution viewed with a scanning electron microscope (SEM). The cross-section was created with a focused ion beam (FIB). To protect the cutting edge, a layer of platinum was added on top of the sample. The structure itself is emphasized because of charging effects at the PDMS material border. For optical enhancement dotted guiding lines were drawn into the cross-section. The TiO_2_ layer is non conductive, therefore features as small as the average particle size (≈21 nm) cannot be seen in the scan. The high index layer can be identified as the dark layer between the structured PDMS substrate and the conductive platinum protection layer on top. It evens out the nanostructure of the substrate and has a thickness of 95 to 200 nm.

The second step to create the flexible photonic crystal membrane is to apply a layer of material with a high refractive index on top of the nanostructure to form a structured waveguide. We used TiO_2_ nanoparticles (titanium(IV) oxide, mixture of rutile and anatase, 33–37 wt % in H_2_O from Sigma-Aldrich, St. Louis, Missouri, USA; diameter < 150 nm; ≈21 nm primary particle size of starting nanopowder). It has a high refractive index of 2.8 (rutile) to 2.5 (anatase) in the visible spectrum at 632 nm. While a continuous evaporated high-index layer forms cracks and gaps under strain, the loosely packed high index nanoparticulate layer offers better strain performance [15]. To control the thickness of the high-index layer different concentrations of nanoparticles are diluted in distilled water, ranging from 2 to 12 wt % in steps of 2 wt %. 14 wt % was also tested but the resonance quality regarding to signal to noise ratio of our sample was too low for meaningful statements. Therefore, all experiments were conducted only with the concentrations below 14 wt %. Furthermore 0.5 wt % of fluorosurfactant (PFT) (NOVEC FC-4430 from 3M) was added. This results in an even lower contact angle between the solution and the PDMS substrate. The mixture is stirred for 5 min (Figure 6b1). The PDMS membranes are clamped to an appropriate spin coating chuck we designed for the specific geometry of our membranes. To avoid ripples in the membranes, the spincoating chuck slightly stretches the membranes. The nanostructured area of the membrane is covered with the nanoparticle solution and the samples are allowed to rest for 30–60 s such that the contact angle reduces due to the PDMS-b-PEO in the substrate and the PFT in the solution. We tested different settings for the spin coating, but for low speeds the resulting surface appearance was not homogeneous. A spin coating program with a ramp of 5000 rpm/s and a final speed of 2000 rpm for 60 s is used for our experiments in this paper for its satisfying results in terms of surface quality. A more detailed study regarding the influence of varying spin coating parameters could lead to interesting results and will be done in future work. Most of the solution is spun off the substrate and from the remaining thin layer the water evaporates within a few seconds. Figure 6b2 and 6b3 illustrate this process. The particles on the surface form an amorphous high index layer over the imprinted nanostructure as depicted in Figure 7. As the nanoparticle layer partially consists of air, the refractive index of this layer is lower than that of a bulk layer of TiO_2_.

### Measurement setup

**Figure 8:**
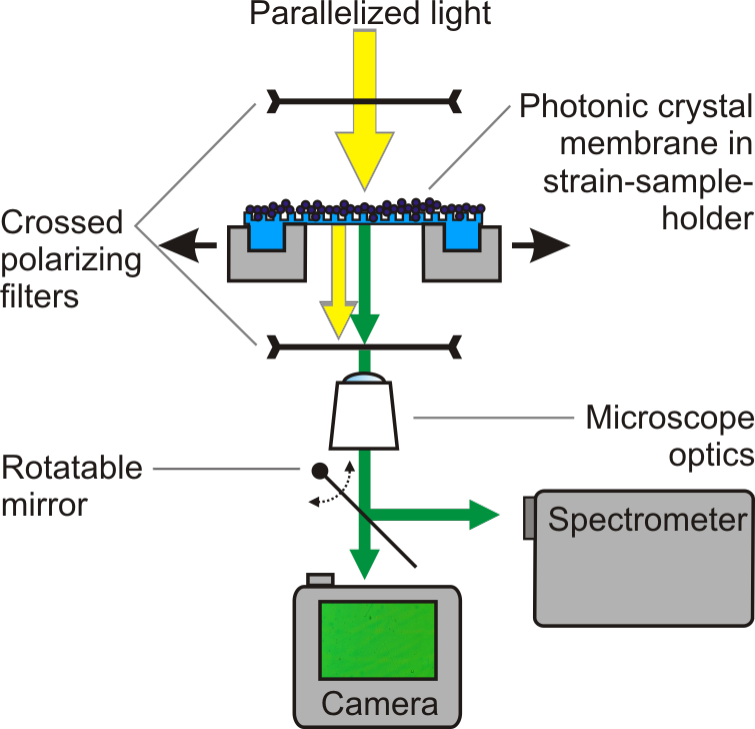
The measurement setup consists of a transmission light microscope with crossed polarization filters before and after the sample for background suppression. The groves of the sample have a 45° angle to both polarization directions. The sample holder allows for an adjustable strain of up to 20%.

The experimental characterization is conducted in a transmission light microscope setup (Figure 8). The parallelized beam of the microscope light source is sent through a polarization filter. Its polarization direction is adjusted to 45° with respect to the sample’s grating direction. The flexible photonic crystal is clamped into a sample holder that allows for adjusting the strain to up to 20% in the direction perpendicular to the grating lines. Below the stretching setup another polarization filter is set to 90° to the first filter’s polarization direction. Thus, only light interacting with the crystal structure passes the polarization setup [20]. The light is then captured by the lens and directed from the microscope either to a spectrometer or a camera. Spectral information and photographs are recorded for all samples and all strain states.

## References

[1] Wang S S, Magnusson R (1993). Appl Opt.

[2] Rosenblatt D, Sharon A, Friesem A A (1997). IEEE J Quantum Electron.

[3] Fan S, Joannopoulos J (2002). Phys Rev B.

[4] Jahns S, Bräu M, Meyer B-O, Karrock T, Gutekunst S B, Blohm L, Selhuber-Unkel C, Buhmann R, Nazirizadeh Y, Gerken M (2015). Biomed Opt Express.

[5] Block I D, Mathias P C, Jones S I, Vodkin L O, Cunningham B T (2009). Appl Opt.

[6] Descharmes N, Dharanipathy U P, Diao Z, Tonin M, Houdré R (2013). Phys Rev Lett.

[7] Yokouchi N, Danner A J, Choquette K D (2003). IEEE J Sel Top Quantum Electron.

[8] Fortes L M, Gonçalves M C, Almeida R M (2011). Opt Mater.

[9] Hsu Q-C, Hsiao J-J, Ho T-L, Wu C-D (2012). Microelectron Eng.

[10] Pradana A, Gerken M (2015). Photonics Res.

[11] Fudouzi H, Xia Y (2003). Langmuir.

[12] Karrock T, Gerken M (2015). Biomed Opt Express.

[13] Privorotskaya N L, Choi C J, Cunningham B T, King W P (2010). Sens Actuators, A.

[14] Foland S J, Lee J-B (2013).

[15] Karrock T, Schmalz J, Nazirizadeh Y, Gerken M (2014). MRS Online Proc Libr.

[16] Liu M, Sun J, Sun Y, Bock C, Chen Q (2009). J Micromech Microeng.

[17] Mata A, Fleischman A J, Roy S (2005). Biomed Microdevices.

[18] Johnston I D, McCluskey D K, Tan C K L, Tracey M C (2014). J Micromech Microeng.

[19] Yao M, Fang J (2012). J Micromech Microeng.

[20] Nazirizadeh Y, Müller J G, Geyer U, Schelle D, Kley E-B, Tünnermann A, Lemmer U, Gerken M (2008). Opt Express.

